# Cellular and molecular responses to acute cocaine treatment in neuronal-like N2a cells: potential mechanism for its resistance in cell death

**DOI:** 10.1038/s41420-018-0078-x

**Published:** 2018-07-17

**Authors:** Ramesh B. Badisa, Sungsool Wi, Zachary Jones, Elizabeth Mazzio, Yi Zhou, Jens T. Rosenberg, Lekan M. Latinwo, Samuel C. Grant, Carl B. Goodman

**Affiliations:** 1 College of Pharmacy and Pharmaceutical Sciences, Florida A&M University, Tallahassee, FL 32307 USA; 2 The National High Magnetic Field Laboratory, Florida State University, Tallahassee, FL 32310 USA; 3 Department of Biomedical Sciences, Florida State University College of Medicine, Tallahassee, FL 32306 USA; 4 Department of Biological Science, Florida A&M University, Tallahassee, FL 32307 USA

**Keywords:** Cell death in the nervous system, Neuroscience

## Abstract

Cocaine is a highly abused drug that causes psychiatric and neurological problems. Its entry into neurons could alter cell-biochemistry and contribute in the manifestation of early pathological symptoms. We have previously shown the acute cocaine effects in rat C6 astroglia-like cells and found that these cells were highly sensitive to cocaine in terms of manifesting certain pathologies known to underlie psychological disorders. The present study was aimed to discern acute cocaine effects on the early onset of various changes in Neuro-2a (N2a) cells. Whole-cell patch-clamp recording of differentiated cells displayed the functional voltage-gated Na^+^ and K^+^ channels, which demonstrated the neuronal characteristics of the cells. Treatment of these cells with acute cocaine (1 h) at in vivo (nM to μM) and in vitro (mM) concentrations revealed that the cells remained almost 100% viable. Cocaine administration at 6.25 μM or 4 mM doses significantly reduced the inward currents but had no significant effect on outward currents, indicating the Na^+^ channel-blocking activity of cocaine. While no morphological change was observed at in vivo doses, treatment at in vitro doses altered the morphology, damaged the neurites, and induced cytoplasmic vacuoles; furthermore, general mitochondrial activity and membrane potential were significantly decreased. Mitochondrial dysfunction enabled the cells switch to anaerobic glycolysis, evidenced by dose-dependent increases in lactate and H_2_S, resulting unaltered ATP level in the cells. Further investigation on the mechanism of action unfolded that the cell’s resistance to cocaine was through the activation of nuclear factor E2-related factor-2 (*Nrf-2*) gene and subsequent increase of antioxidants (glutathione [GSH], catalase and GSH peroxidase [GPx]). The data clearly indicate that the cells employed a detoxifying strategy against cocaine. On a broader perspective, we envision that extrapolating the knowledge of neuronal resistance to central nervous system (CNS) diseases could delay their onset or progression.

## Introduction

The abuse of pharmacological substances in modern society has increased exponentially during the last decade and has led to burgeoning psychiatric problems worldwide^1^. Cocaine is one of the widely abused drugs that rapidly crosses the blood brain barrier and binds to various plasma membrane transporters on neurons. Even though cocaine is degraded rapidly from the body as evidenced by its short half-life^2–4^, its brief stay in the CNS sets off profound psychostimulatory impact on its users. In addition, cocaine also exerts toxic effects in different CNS cells, such as astrocytes, under in vivo and in vitro situations with regards to altering the morphology or cell size^5^ or manifesting certain pathology underlying psychological disorders^6^. So far, there is no clear understanding on how cocaine exerts cytotoxicity in neurons.

Autophagy^7^ and oxidative stress arising from dopamine degradation at the synaptic cleft^8^ have been cited as the common causes of death. Owing to cocaine’s rapid degradation and clearance from the CNS^2–4^, and reversible nature of autophagy^6,9^, the role of autophagy in neuronal toxicity still remained unclear. Cocaine administration at 50–450 mg/kg/day in animal models neither caused cell death nor induced neurodegenerative changes^10,11^ which raised doubt on the role of oxidative stress in neuronal death. Consistent with these reports, in vitro studies in mouse neuro-2a (N2a) cells treated with milli molar doses of cocaine also did not show cell death (unpublished results). In case of humans, post-mortem examination of several long-term cocaine users revealed only a 16% neuronal loss in the striatum and mid-brain^12^.

Compared to the long-term cocaine abuse^12^, a mere 16% neuronal loss, though significant, is not considered drastic, and highly bewildering given the perception that in vivo neurons are highly sensitive to cocaine-induced oxidative stress arising from dopamine degradation^8^. Although age, gender, esterase levels, and genetics are known contributing factors that may control neuronal damage in the brain, absence of drastic neuronal destruction in the long-term cocaine users^12^ or failure of neuronal casualty in animal studies^10,11^ may thereby imply the presence of some unknown factors resisting cocaine detrimental effect in the CNS. Lack of severe neuronal loss as a common denominator under in vivo^10–12^ and in our in vitro study is neither a co-incident nor a farfetched idea but may denote a similar underlying mechanism of neuronal resistance that warrants further investigation. In addition, identification of biochemical changes in cells with cocaine exposure is also important to determine its disruptive affects—a criterion in translational research.

It is not yet known what biochemical changes would cocaine induce in neurons; nor is clear how the change in neuronal biochemistry could lead in the development of resistance to cocaine. We hypothesized that identification of early response-changes with cocaine treatment would reveal the sub-cellular targets in neurons, and this identification would provide mechanistic insights underlying the cellular resistance to cocaine. The early responses we focused include change in gross cell morphology, cell viability (at in vivo and in vitro concentrations), vacuolation, cytotoxic markers that compromise cell membrane integrity (lactate dehydrogenase [LDH], reactive oxygen species [ROS], and lipid peroxidation), biochemical markers (general mitochondrial activity, its membrane potential, lactate, hydrogen sulfide [H_2_S]), and ATP levels. Finally, we investigated the genes responsible for cellular resistance to acute cocaine treatment and measured the levels of antioxidants (GSH, catalase and GPx) in the cells. Understanding the mechanism of neuronal resistance may carry significance in the field of neuroscience because it could help identifying the neuroprotective factor/s responsible for resistance and explore its application to several CNS diseases in terms of delaying their onset or progression.

## Results

### Effect of cocaine at in vivo and in vitro doses

**Fig. 1:**
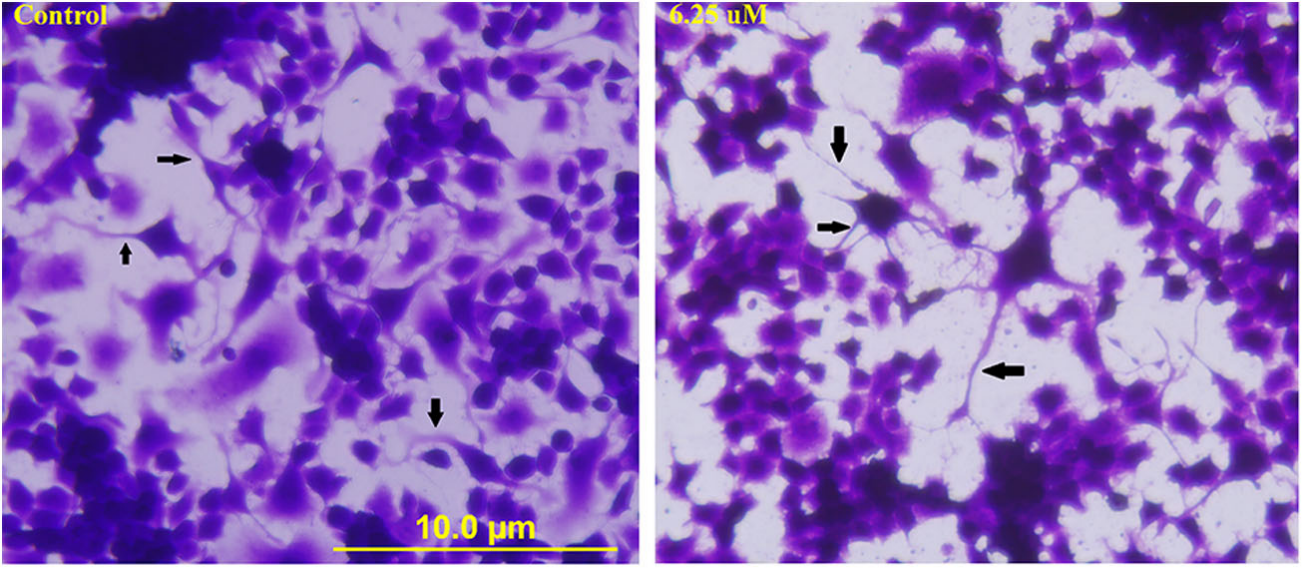
Effect of in vivo concentration of cocaine on morphology. The cells were treated with equal volume of vehicle (PBS control) or 6.25 μM cocaine for 1 h. Morphological images were taken using an inverted phase contrast IX-70 Olympus microscope with ×20 objective. Arrows show the inter-neuronal connections in the control and 6.25 μM treated cells. Scale bar: 10 μm

After confirming the neuronal-like characteristics of N2a cells (Supplementary Figures S1, 2), we next assessed the effect of cocaine on neurite-connections and cell viability at pharmacologically relevant in vivo doses, which usually range from nano to lower micro molar in drug addicts^13^. Under our test conditions, none of the doses treated for 1 h (1, 5, 10, 25, and 50 nM; and 0.1, 0.2, 0.5, 1, 5, and 6.25 µM) induced any type of changes in the cells. Even at 6.25 µM treatment, the cells appeared as the controls with well-preserved neurites and inter-neuronal connections (Fig. 1, arrows in 6.25 µM). Similarly, there was no loss (*n* = 8, *P* *>* 0.05) in cell viability at any of these doses compared to the control (Supplementary Figure S3A). This warranted increasing of cocaine to milli molar concentrations based on previous reports^14–16^.

**Fig. 2:**
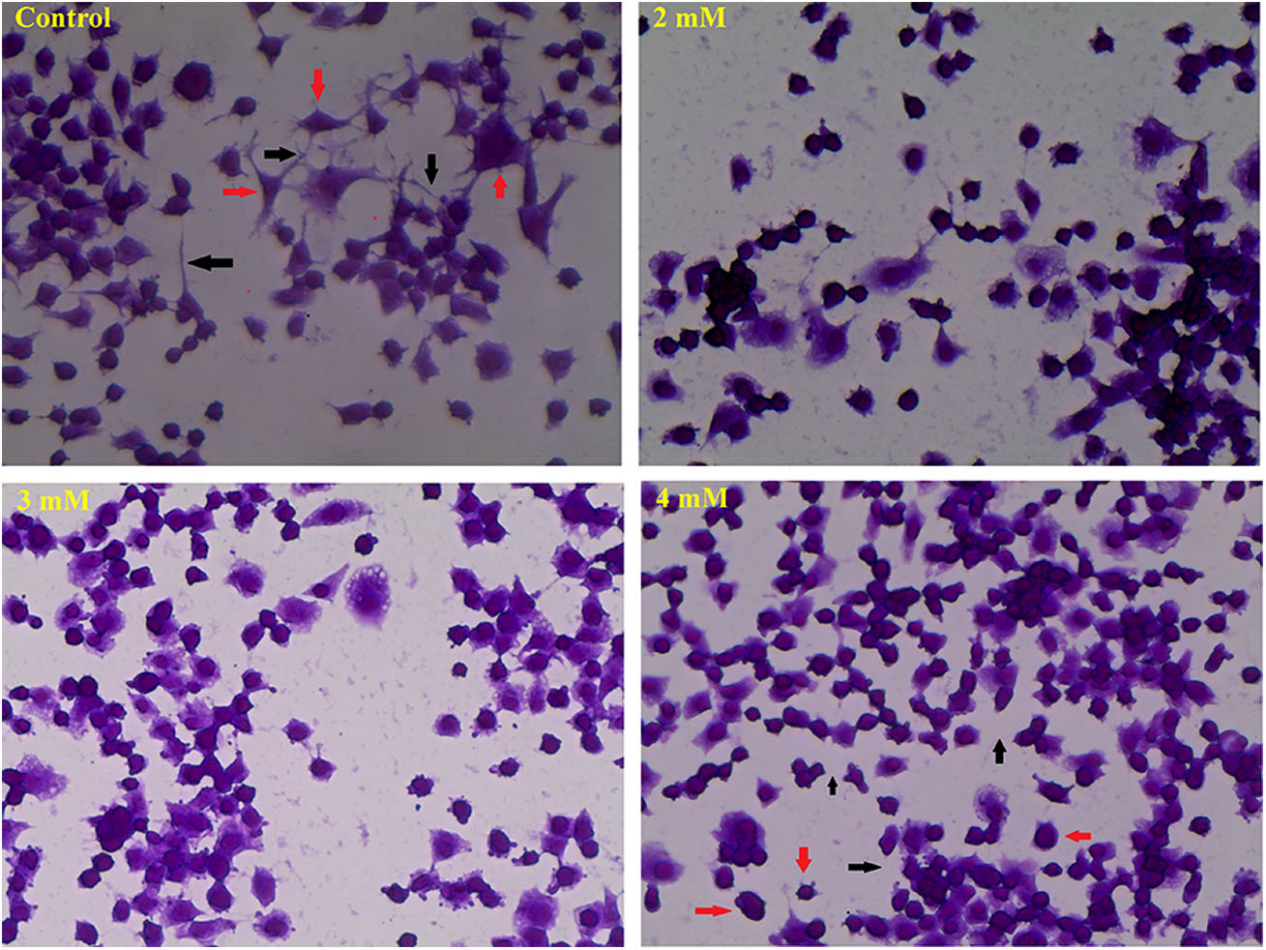
Effect of in vitro doses of cocaine on cell morphology. The cells were treated with equal volume of vehicle (PBS control) or 2–4 mM cocaine for 1 h. Morphological images were taken using EVOS Cell Imaging Systems with ×40 objective. Black arrows show the inter-neuronal connections in the control cells and their loss with 4 mM treatment; red arrows indicate triangular or polygonal morphology of control cells but changed to round upon 4 mM treatment.

**Fig. 3:**
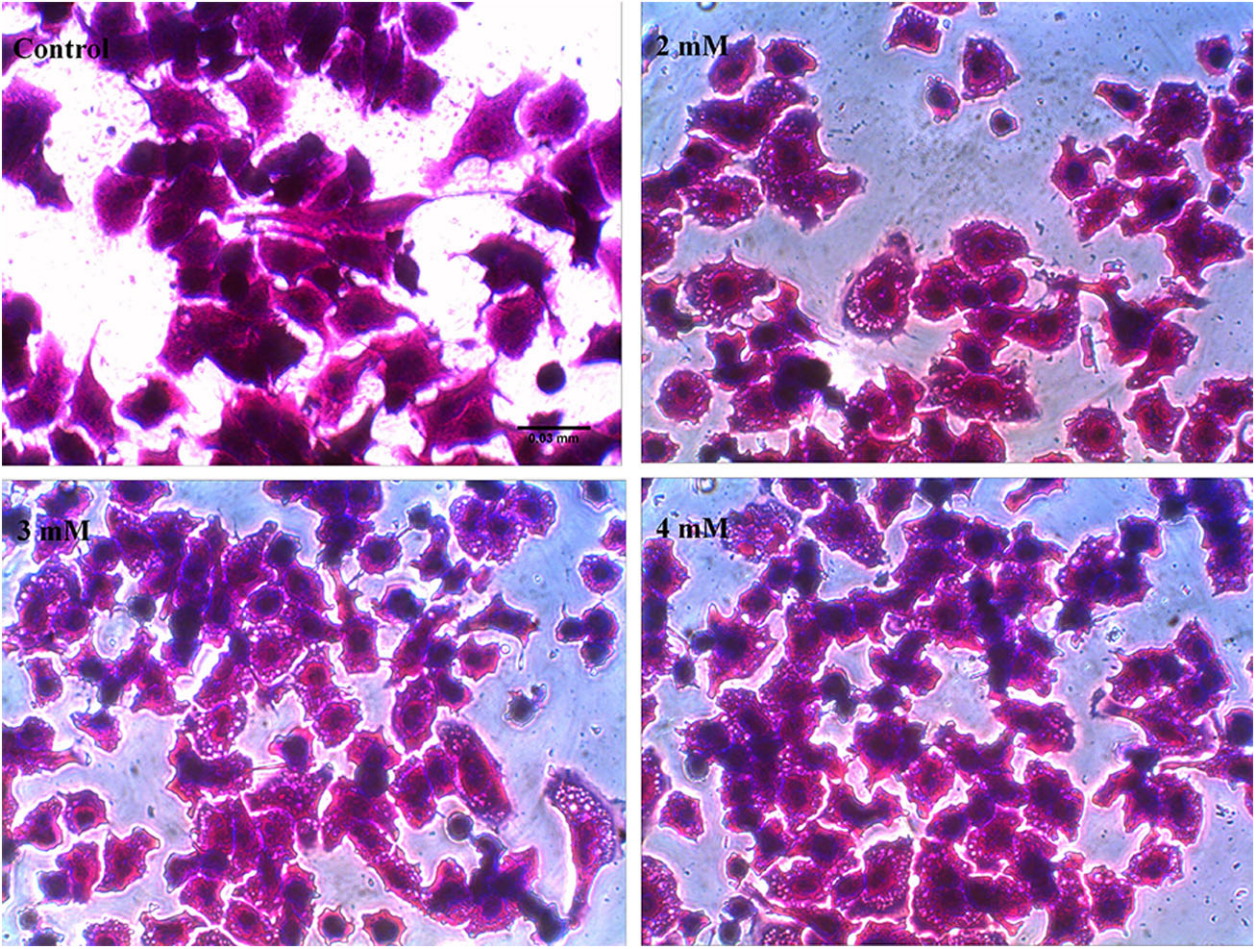
Induction of vacuoles in cells at in vitro doses of cocaine. The cells were treated with equal volume of vehicle (PBS control) or 2–4 mM cocaine for 1 h. Optical images of vacuoles were taken using an inverted phase contrast IX-70 Olympus microscope with ×40 objective. Scale bar: 0.03 mm

Alteration in cell morphology is one of the earliest signs of toxicity; so we initially evaluated the effect of 2, 3 and 4 mM cocaine on cell morphology. While control cells displayed triangular or polygonal morphology (Fig. 2, red arrows), cocaine treatment at 2–4 mM significantly altered the morphology to round (Fig. 2, red arrows in 4 mM treatment). Furthermore compared to the control, neurite structures were damaged with treatment, causing loss in the inter-neuronal connections (Fig. 2, black arrows in 4 mM); yet, there was no change in cell viability at any treatment (Supplementary Figure S3B; *n* *=* 12, *P* *>* 0.05). However, cytoplasmic vacuoles were observed (Fig. 3) in a dose-dependent manner (Supplementary Figure S3C; *n* = 12, *F*_(3,44)_ = 9.49, *P* *<* 0.05) in cocaine-treated cells compared to the control. Vacuolation with cocaine treatment was also observed earlier in rat C6 astroglia-like cells;^6^ however, unlike N2a neuronal-like cells, cocaine cytotoxicity was more pronounced in C6 astroglia-like cells;^6^ similarly, the morphological alterations were more rapid in these cells^6^ compared to the N2a neuronal-like cells. Videography in the present study indicated that the morphological changes began manifesting after about 30 s of cocaine treatment (3 mM) in rat C6 astroglia-like cells followed by a type of movement -referred as “retractory movement”. At the end of the movement, the cells were seen clumped in the form of separate islands (Supplementary Video 1). Interestingly, N2a neuronal-like cells treated with 3 mM cocaine did not exhibit the retractory movement (Supplementary Video 2) even up to 5 min of videography. Although the mechanism for “retractory movement” observed in C6 astroglia-like cells is not known, nor investigated currently, it is possible that actin filaments of the cytoskeleton^6^ and calcium ions are involved. The aim of these videos is only to complement the resistant nature of N2a neuronal-like cells to cocaine treatment compared to C6 astroglia-like cells^6^ in terms of retractory movement.

**Fig. 4:**
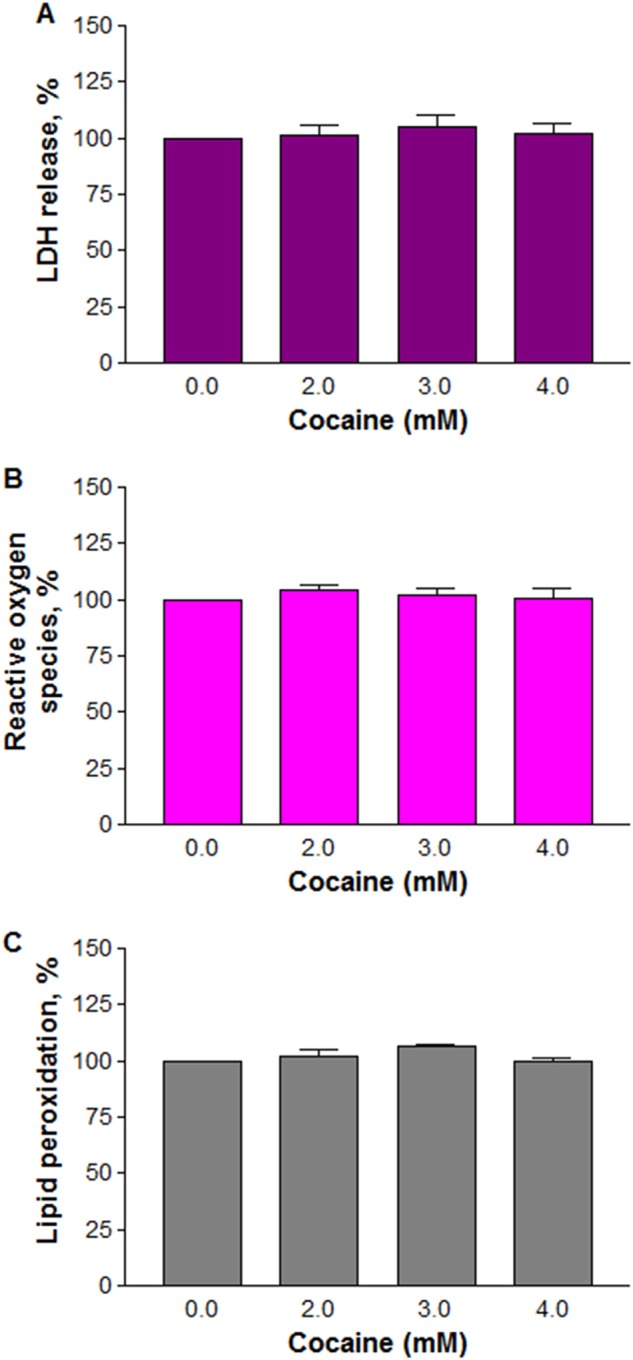
Effect of cocaine on plasma membrane integrity. The cells were treated with various concentrations of cocaine for 1 h. LDH (**a**) release (*n* *=* 8), ROS (**b**) generation (*n* *=* 8) or lipid (**c**) peroxidation (*n* *=* 12) were measured in a micro plate reader. Data were represented as mean ± SEM, *P* > 0.05, insignificant compared to control, one-way ANOVA, Dunnett’s multiple comparison test

Despite treatment of N2a neuronal-like cells with milli molar doses of cocaine, there was neither LDH release to denote membrane damage (Fig. 4a, *n* = 8; *P* *>* 0.05) nor ROS generation (Fig. 4b; *n* = 8, *P* *>* 0.05) or lipid peroxidation (Fig. 4c; *n* = 12, *P* *>* 0.05), suggesting their non-involvement in the altered cell morphology.

### Inhibition of sodium channel activity

**Fig. 5:**
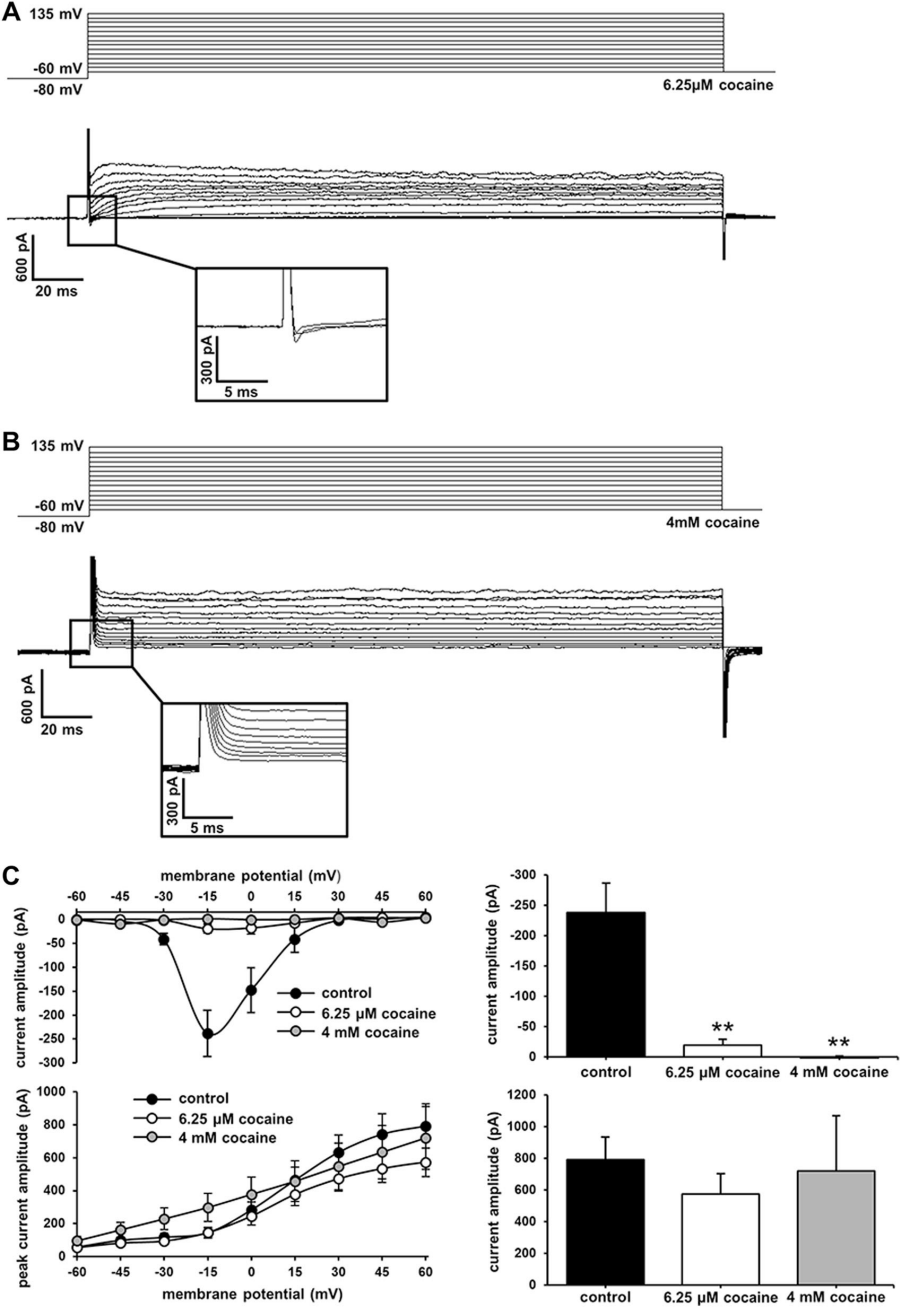
Cocaine treatment reduces inward current amplitude. Representative voltage clamp trace showing reduced inward currents after **a** low dose (6.25 μM) and **b** high dose (4 mM) cocaine treatment. Step size = 15 mV. The inset shows a high magnification view of inward currents. **c** (Left) Current–voltage relationship of inward currents (top) and outward currents (bottom) measured from untreated, low dose, and high dose cocaine-treated N2a cells (*n* = 8 for each group). **c** (Right) Average peak current amplitude at −15 mV (top, inward currents) and +60 mV (bottom, outward currents). **P < 0.001, significant in comparison to corresponding controls, one-way ANOVA, Bonferroni’s multiple comparison tests

To examine the effects of cocaine on electrophysiology, the cells were submerged in low (6.25 μM) or high (4 mM) doses of cocaine in extracellular recording solution prior to whole-cell patch-clamp recording. Cocaine treatment significantly reduced inward currents at both low (Fig. 5a) and high (Fig. 5b) doses (*n* = 8, *F*_(2,21)_ = 21.81, *P* < 0.001) and had no significant effect on outward currents (Fig. 5c). The peak inward current amplitude at −15 mV was −238.37 ± 48.29 pA in untreated cells compared to −19.26 ± 9.61 pA in low cocaine dose and 1.61 ± 2.55 pA in high cocaine dose. This result is consistent with the known Na^+^ channel-blocking activity of cocaine in excitable cells^17,18^. No differences were observed between untreated and cocaine-treated cells in spike activity or spontaneous postsynaptic current activity (Supplementary Figure S4).

### Mitochondrial dysfunction

**Fig. 6:**
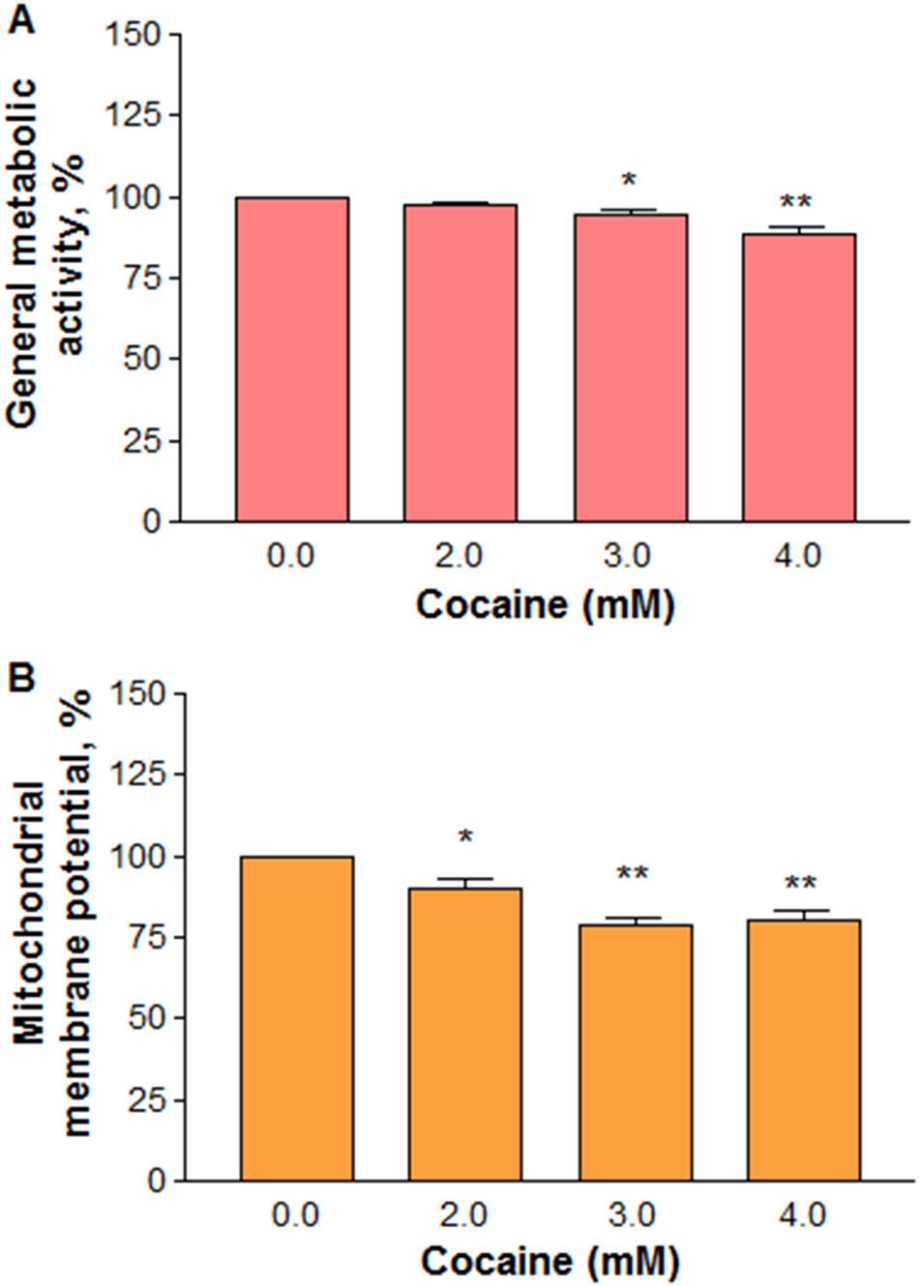
Effect of cocaine on mitochondria. The cells were treated with various concentrations of cocaine for 1 h. General metabolic activity (**a**, *n* *=* 12) or membrane potential (**b**, *n* = 8) were measured in a micro plate reader. Significant in comparison to the control (*P *<* 0.05 or **P < 0.01, one-way ANOVA, Dunnett’s multiple comparison test)

The mitochondrial enzymes reduce the tetrazolium compound of MTS into a pink formazan^19^. Thus, measurement of total formazan in cells reflects the general metabolic status of mitochondria. In our study, cells treated with cocaine at 2–4 mM for 1 h showed a small but significant (*n* = 12, *F*_(3,44)_ = 12.17, *P* < 0.05) decrease in the general metabolic activity of mitochondria compared to the control (Fig. 6a). The decrease was (±standard error of the mean (SEM)) 97.4 ± 0.7, 94.5 ± 1.2, and 88.6 ± 2.4% of the control value (100%) at 2, 3, and 4 mM cocaine, respectively. Similarly, there was a significant (*n* = 8, *F*_(3,28)_ = 16.46, *P* *<* 0.05) dose-dependent decrease in membrane potential compared to the control (Fig. 6b). The average decrease (±SEM) at 2, 3 and 4 mM cocaine was 90.0 ± 2.6, 79.1 ± 2.3, 80.4 ± 3.2%, respectively compared to the control (100%).

### Anaerobic features

**Fig. 7:**
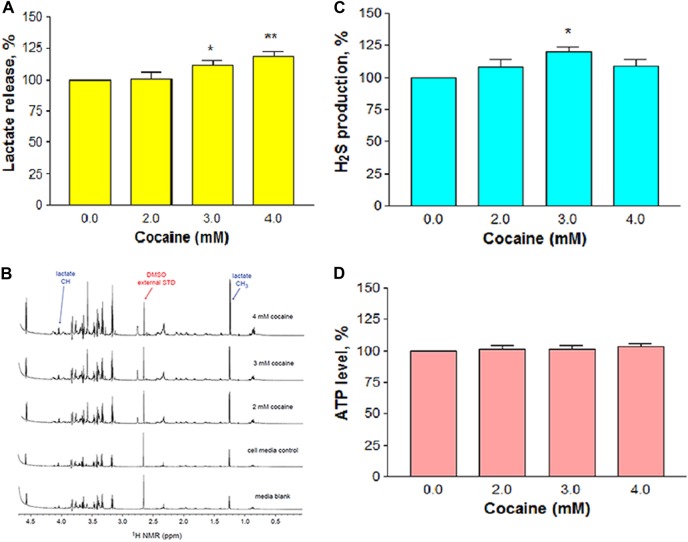
Cocaine-induced anaerobic characters. The cells were treated with various concentrations of cocaine for 1 h. Lactate was measured by colorimetric (**a**) method (*n* = 12). Medium from the untreated cells was taken as a control while the medium without cells was used as a blank. Confirmation of lactate release by^1^H^+^ NMR (**b**) spectroscopy. Lactate peaks are visible in the spectra at 1.25 ppm for the methyl group (doublet) and 4.04 ppm for the methine group (quartet), **c** H_2_S production (*n* = 16), and **d** ATP measurement by bioluminescence (*n* = 8). Significant in comparison to the control (*P *<* 0.05 or **P < 0.01, one-way ANOVA, Dunnett’s multiple comparison test)

Loss in mitochondrial function switches the cells to anaerobic respiration as a means of survival. We evaluated the anaerobic characteristics by measuring the levels of lactate, and H_2_S. Exposure of N2a neuronal-like cells to cocaine at increasing concentrations (2, 3, and 4 mM) for 1 h significantly (*n* = 12, *F*_(3,43)_ = 21.04, *P* *=* 0.0001) and dose-dependently increased lactate release into the medium compared to the control cells (Fig. 7a). The increase (±SEM) was 111.7 ± 3.1% and 118.4 ± 3.9% compared to the control (100%) at 3 and 4 mM cocaine, respectively. Cocaine at 2 mM or less did not induce significant lactate release in the medium. In order to further confirm the release of lactate, the study was repeated with 2–4 mM cocaine treatments and the media were analyzed by ^1^H^+^ NMR spectroscopy. After suppressing the dominant water peak at 4.8 ppm in the spectrum by applying WATERGATE pulse sequence^20^, the relevant frequency range (0–4.7 ppm) of each spectrum was shown (Fig. 7b). Lactate peaks were visible at 1.25 ppm for the methyl group (doublet) and 4.04 ppm for the methine group (quartet). Since there was no cocaine peak detected in the experimental blank, it is obvious that approximately a 3-fold increase in lactate compared to the control cells (Fig. 7b) was due to cocaine treatment alone.

We next tested whether cocaine treatment could release H_2_S, a biomarker of anaerobic metabolism, from the cells. The data indicated that there was a small but significant (*n* = 16, *F*_(3,84)_ = 3.57, *P* *<* 0.05) increase in H_2_S release in cocaine treated cells compared to the control (Fig. 7c). The increase (±SEM) was 108.2 ± 5.8%, 119.9 ± 3.7%, and 108.7 ± 5.2% compared to the control (100%) at 2, 3, and 4 mM cocaine, respectively. Hypoxic state did not alter the ATP level (*n* = 8, *P* *>* 0.05) in cocaine-treated cells (Fig. 7d).

### Cell survival with cocaine treatment was not associated with *survivin* (Birc5) gene

**Fig. 8:**
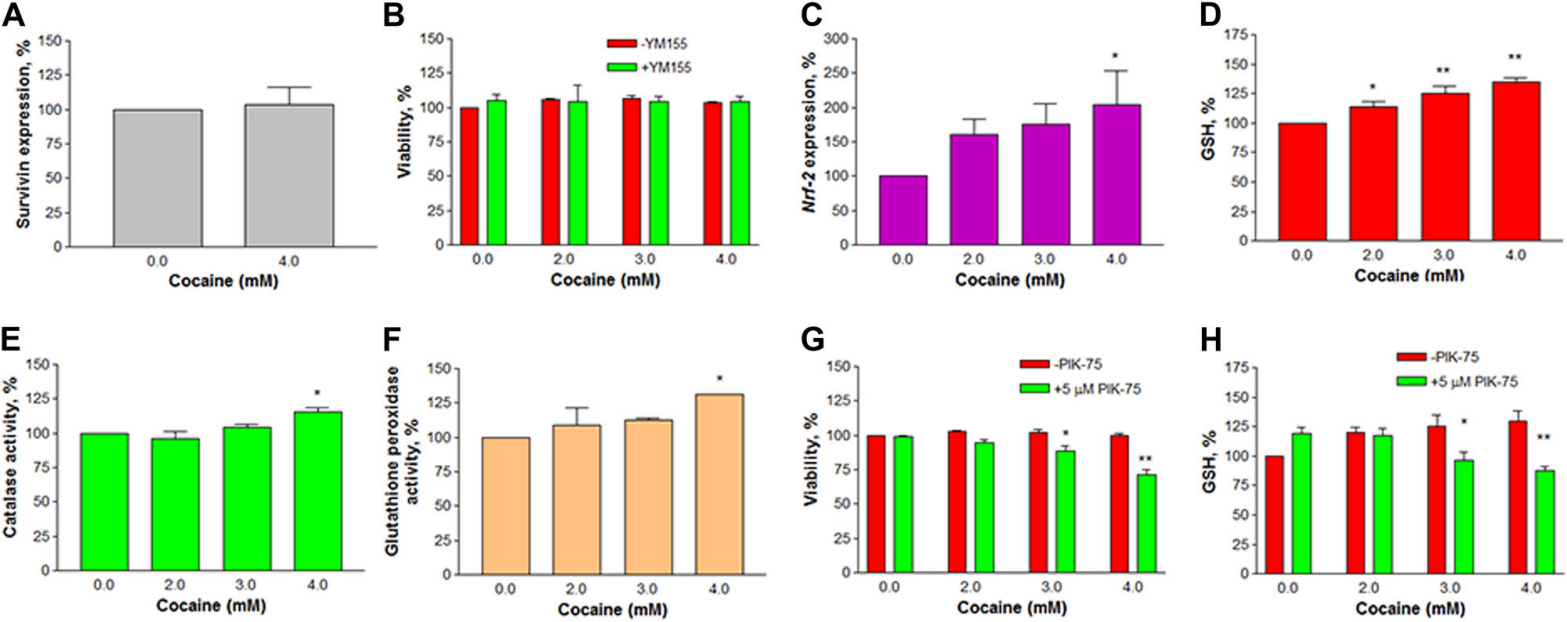
Effect of cocaine on *survivin*, *Nrf-2*, and antioxidants. For *survivin* gene expression, the mRNA levels in 4 mM cocaine-treated and control cells were quantified (*n* = 4) by real-time PCR using *GAPDH* as the reference gene (**a**); in another study, the cells were pretreated with 1 µM YM155 (*survivin* gene inhibitor) for 30 min, followed by cocaine co-treatment for 1 h, and the cell viability was measured (*n* = 4) in a micro plate reader (**b**). For *Nrf-2* gene expression, the mRNA level was quantified (*n* *=* 3) by real-time PCR using *GAPDH* as the reference gene (**c**). Colorimetric assays were performed for glutathione (*n* = 9) (**d**) or catalase (**e**, *n* = 3) or GPx (**f**, *n* = 3) or role of PIK-75 (*Nrf-2* inhibitor) on cocaine treated cells for viability (**g**, *n* = 8) or glutathione (**h**, *n* = 8). Data were represented as mean ± SEM, significant compared to the control (*P *<* 0.05 or **P < 0.01, one-way ANOVA, Dunnett’s (**a**, **c**, **d**–**f**) or Bonferroni’s (**b**, **g**, **h**) multiple comparison tests)

Because there was no cell death with cocaine treatment at in vitro concentrations, we investigated whether *survivin*, a member of the anti-apoptotic family of genes^21^, was associated with survival of the cells upon cocaine treatment. Since cell viability was not changed at 4 mM cocaine treatment compared to the control, we measured *survivin* gene expression only at this dose. There was no significant difference in *survivin* expression in cocaine treated cells compared to the control (Fig. 8a). To further confirm the result, we pre-treated the cells with 1 µM YM155, a *survivin* inhibitor, for 30 min, followed by cocaine treatment (2–4 mM) for 1 h. There was no change (*n* = 4, *P* *>* 0.05) in cell survival with cocaine treatment in the presence or absence of YM155 inhibitor (Fig. 8b). Pre-incubation of cells with 1 µM YM155 for 24 h, followed by cocaine (2–4 mM) co-treatment for 1 h also did not cause cell death (data not shown). These results indicate clearly that the observed neuronal survival with cocaine treatment was not associated with *survivin* gene.

### Cocaine treatment activated *Nrf-2* expression and increased antioxidants

Previous reports showed that H_2_S release was associated with activation of nuclear factor E2-related factor-2 (*Nrf-2*) as a response to stress^22^. We investigated whether cocaine-induced H_2_S release (Fig. 7c) could activate *Nrf-2* gene expression in N2a neuronal-like cells with cocaine treatment. It was found that there was a significant (*n* = 7–12, *F*_(3,47)_ = 2.5, *P* < 0.05) upregulation of *Nrf-2* gene expression compared to the control (Fig. 8c). The increase was (±SEM) 203.8 ± 50.3 of the control value (100%) at 4 mM. Since *Nrf-2* is known to increase several antioxidant systems^23^, we then measured three antioxidants, namely GSH, catalase, and glutathione peroxidase in cocaine-treated cells. It was found that cocaine treatment caused a significant (*n* = 12, *F*_(3,44)_ = 15.68, *P* *<* 0.01) increase in GSH level compared to the control (Fig. 8d). The GSH levels were (±SEM) 114.19 ± 4.5%, 125.58 ± 6.0%, and 134.71 ± 3.4% of the control value (100%) at 2, 3, and 4 mM cocaine, respectively. Similarly, there was a significant increase in catalase [*n* = 3, *F*_(__3,8__)_ = 7.04, *P* < 0.05] and GPx activities [*n* = 3, *F*_(3,8)_ = 4.35, *P* < 0.05] at 4 mM cocaine treatment compared to the corresponding controls (Fig. 8e, f).

### *Nrf-2* inhibition caused cell death through decreased GSH

Because cell resistance to high doses of cocaine in our study was due to increased antioxidants through *Nrf-2* activation (Fig. 8c–f), we reasoned that inhibition of *Nrf-2* should decrease the level of antioxidants and consequently decrease the cell viability with cocaine treatment. To prove this, we pre-treated the cells with 5 µM PIK-75 [an inhibitor of *Nrf-2*]^24^ for 30 min, followed by cocaine (2–4 mM) co-treatment for 1 h. PIK-75 alone did not cause cell death (*n* = 8, *P* > 0.05); however, with cocaine co-treatment for 1 h, it caused significant cell death (*n* = 8, *F*_(__7,56__)_ = 19.34, *P* < 0.001). For example, the viability was decreased to 94.8, 88.3 and 71.6% at 2, 3, and 4 mM cocaine, respectively compared to the control cells (Fig. 8g). Under this condition, we measured the GSH level in the cells. While PIK-75 alone caused significant increase (119.2 ± 5.1%) in GSH level compared to the untreated control (100%, *n* = 8, *P* < 0.05), cocaine co-treatment for 1 h caused significant decrease in the GSH level compared to cocaine-alone-treated group (*n* = 8, *F*_(__7,56__)_ = 6.352, *P* < 0.001). For example, the GSH level was decreased to 117.2 ± 6.0%, 96.7 ± 6.6%, and 87.7 ± 3.8% at 2, 3, and 4 mM cocaine, respectively (Fig. 8h). The decreased GSH level with PIK-75 pre-treatment corresponded with the decreased cell viability. These results prove the involvement of *Nrf-2*-dependent increase of antioxidants for cell resistance against cocaine.

## Discussion

Besides identifying several early response-changes, the report on the lack of cell death to high cocaine doses in N2a neuronal-like cells was a new observation unknown previously. Assessment of early response-changes under in vivo situation would have been relevant to decipher cocaine action, but direct understanding of those changes is impeded due to body complexity. Cell cultures, on the other hand, offer unique opportunity to explore the mechanism of response under controlled environment, a factor responsible for using in vitro model in this study. Employing primary cultures here is not practical on account of restricted growth potential, finite life span and lack of cell homogeneity. The N2a cells employed in this study have neuronal origin, and upon differentiation functioned as neurons;^25,26^ our results corroborated these reports in terms of morphology (Supplementary Figure S1) and function (Supplementary Figures S2, 4).

The use of higher cocaine doses in our study became imperative owing to lack of effect on morphology (Fig. 1) or viability (Supplementary Figure S3A) at lower concentrations. This necessitated increasing of cocaine concentrations several fold. Under in vitro studies, cocaine concentrations have extended into milli molar range^14,15,27,28^. Thus testing 8.8^29^, 10^14,28,30^, or 13 mM^31^ cocaine at even longer-incubation intervals despite cocaine’s relatively short half-life of ~1 h^2–4^ are not atypical. The highest concentration (4 mM) in our study was 2.2–3.25 times lower compared to 8.8^29^ and 13 mM^31^ cocaine, respectively. Therefore, cocaine concentrations tested in our study were in the acceptable range for in vitro research. The use of higher concentrations of cocaine in our study was compared with in vivo administrative and clinical doses, and was explained in detail in the Supplementary results. In spite of high doses, the cells remained viable to acute (1 h) cocaine treatment (Supplementary Figure S3B). A time-course of study might have revealed loss in the cell viability, as shown in a recent report^32^; however, we did not undertake such a study currently because the intension of acute treatment for 1 h was to mimic the maximum period of euphoria in cocaine addicts, which usually wears off within 1 h for typical amounts and routes of intake^33^, and to evaluate various early response-changes within this period because it assists in knowing early sub-cellular targets of cocaine in neurons.

We found that mitochondria were the primary targets of cocaine (Fig. 6a, b). Interestingly, dysfunctional mitochondria (Fig. 6b) did not pose threat to viability of the cells because they survived through anaerobic respiration as evidenced by lactate release (Fig. 7a, b). Since cocaine is cleared off rapidly from the body owing to its short half-life^2–4^, it is possible that one-time cocaine abuse by humans, yielding the pharmacological doses found in the brain^13^, may not affect neurites outgrowth or neuronal viability. Consistence with these observations was the results from animal models with short-term cocaine administration of 50 to 450 mg/kg/day that neither caused cell death nor induced neurodegenerative changes^10,11^. However, as addicts consume cocaine frequently, it is possible that the cells are affected in the long-run. Echoing these views is the post-mortem examination of several long-term cocaine addicts which showed loss of dopaminergic neurons in striatum and midbrain^12^.

Consistent with earlier reports^9,34–37^, acute cocaine treatment of cells resulted in cytoplasmic vacuolation (Fig. 3; Supplementary Figure S3C). In spite of it, lack of concomitant increase in cocaine toxicity to cells (Supplementary Figure S3B) implies that the formation of vacuoles in these cells was a secondary cause to the toxicity, while in C6 astroglia-like cells, vacuolation appeared as one of the primary events to toxicity^6^. Lack of cell death to acute cocaine treatment in our study is not only in contrast with the general perception of neurons being sensitive to cocaine but also contradicted with previous reports in neuronal cultures^14,38–40^. Variations in culture conditions and incubation periods [e.g., 1 h acute exposure vs. 2–4 days chronic exposure^14,38–40^] are some possible causes of this discrepancy.

Despite hypoxic state (Fig. 7a, b), there was no change in ATP level in cocaine treated cells (Fig.7d). Production of H_2_S (Fig. 7c) indicated that the unchanged ATP level in treated cells might have resulted through H_2_S association as shown earlier^41^. Studies also showed that H_2_S production was involved with the activation of transcription factor *Nrf-2* in response to cellular stress^22^. Coinciding with this report, an up-regulation of *Nrf-2* gene was observed in our study with cocaine treatment (Fig. 8c), suggesting that cocaine exposure triggered the stress signals. In support of *Nrf-2* protection through antioxidant system as reported earlier^42,43^, an upregulation of *Nrf-2* gene with cocaine treatment was correlated with increased antioxidants (Fig. 8d–f), while their decrease by the treatment of *Nrf-2* inhibitor (PIK-75) decreased the cell viability with cocaine treatment (Fig. 8g). Because pre-treatment of cells with the inhibitor of *survivin* (YM155) did not cause cell death with cocaine (Fig. 8a, b), it is obvious that the mechanism of cell resistance to cocaine was not of general type; instead, a specific detoxifying strategy through *Nrf-2* gene was responsible for cellular resistance against cocaine treatment. Thus, identification of early response-changes with cocaine treatment indeed revealed that the mitochondria were the main sub-cellular targets in the cells, and provided the insights that *Nrf-2* gene activation was the underlying mechanism for cellular resistance. This supported our hypothesis.

CNS disorders like Parkinson’s disease or Alzheimer disease^44^ or schizophrenia are associated with progressive neuronal loss in the brain. Attempts to cure these diseases were not successful so far. While efforts of curing various CNS diseases are good, their prevention is much better. One of the safest ways to prevent CNS diseases is by achieving neuronal resistance through intracellular regulation. Even though there was no direct relevance of our study to neurodegenerative disorders, we attempted to extrapolate the concept of neuronal “resistance” (lack of cell death) observed in our study to CNS disorders.

For instance, the knowledge on factors responsible for resisting neuronal cell death may be exploited in delaying the onset or progression of neurodegenerative disorders through intracellular regulation. Such feasibility was demonstrated both in vivo and in vitro situations. Studies showed that up-regulation of DJ-1 gene prevented the progression of Parkinson’s disease in animal models^45^. In a similar approach under *in vitro* condition, we showed the resistance of cells to toxins (MPP^+^ and cocaine) through intracellular regulation^6,46^. These studies clearly demonstrated the practical side of achieving cell resistance to toxins [MPP^+6^ and cocaine^46^] or [Parkinson’s^45^ or Alzheimer’s^47^] diseases through intracellular regulation. Thus, achieving neuronal resistance through intracellular (up/down) regulation of some gene/s (e.g., *Nrf-2*) by pharmacological boosting could protect and delay the onset or progression of neurodegenerative disorders. We guesstimate that this approach may facilitate a novel therapeutic strategy for CNS diseases in future.

## Materials and methods

### Materials

RPMI 1640, FBS, penicillin/streptomycin sulfate, amphotericin B, PBS, and l-glutamine, were purchased from Media Tech (Herndon, VA). Crystal violet, rhodamine 123 (Rh 123), L glutaraldehyde, trypan blue, cocaine-HCl (Ecgonine methyl ester benzoate, MW: 339.8), 2′,7′–dichlorofluorescin diacetate (H_2_DCFDA), 5,5-dithiobis-2-nitrobenzoic acid (DTNB), nicotinamide adenosine dinucleotide phosphate (NADPH) and ethylene diamine tetraacetic acid (EDTA) were supplied by Sigma Chemical Company (St. Louis, MO). The *Nrf-2, survivin* (Birc5), and *GAPDH* oligonucleotide primers were obtained from Eurofins Genomics (Louisville, KY). PIK-75 hydrochloride (inhibitor of *Nrf-2*) was purchased from Santa Cruz Biotechnology, Inc. (Dallas, TX). *Survivin* inhibitor YM 155 was obtained from Calbiochem (San Diego, CA). All other routine chemicals were of analytical grade.

### Cell culture

The N2a cells (CCL-131, ATCC) were maintained as a monolayer culture^48^, and has been widely employed for studies on substances of abuse^14,49,50^ or CNS disorders like Parkinson’s^51^ or Alzheimer’s diseases^47,52,53^. One advantage of this cell line is that the fine morphological changes due to any chemical exposure can easily be detected on account of their larger cell size. Unless otherwise specified, all experiments in our study were carried out in phenol red free RPMI-1640 medium containing 10% FBS. Post-seeded cells in culture plates or dishes were allowed to grow 4–5 days in the incubator for spontaneous differentiation of neurite outgrowths. All studies were repeated at least twice.

### Morphology

For gross cell morphological evaluations, crystal violet stained cells^37^ were photomicrographed using EVOS Cell Imaging Systems with ×40 objective. In some studies, morphology or vacuoles of crystal violet-stained cells were taken using an inverted phase contrast IX-70 Olympus microscope. Neurite outgrowths were quantified using image J software (National Institutes of Health).

### Electrophysiology

Whole-cell patch clamp was used to record from N2a cells cultured on plastic cover slips. Cover slips were washed three times with extracellular recording solution containing (in mM) 145 NaCl, 2 KCl, 2 CaCl_2_, 2 MgCl_2_, 10 glucose, and 10 HEPES (312 mOsm, pH 7.4) and were incubated in this solution at room temperature. Cover slips were either left untreated or treated with 6.25 μM or 4 mM cocaine directly in the extracellular solution during recording. Glass electrodes (resistance 1–5 MΩ) were filled with intracellular solution containing (in mM) 130 KCl, 2 NaCl, 10 HEPES, and 5 EGTA (292 mOsm, pH 7.4). Cells were visualized under phase contrast with a Nikon Eclipse Ti-U inverted microscope and attached DS-Qi1 monochrome digital camera.

Recordings were made with an Axopatch 200B amplifier (Molecular Devices, CA) and digitized with a Digidata 1440A system (Molecular Devices, CA). Ionic currents were recorded under a voltage clamp protocol (−60 to 135 mV in 15 mV steps, 250 ms in duration). Spontaneous postsynaptic currents were recorded under continuous voltage clamp at −80 mV for 2 min. A current clamp protocol (−100 to 200 pA in 20 pA steps, 800 ms in duration) was used to assess the ability of N2a cells to fire action potentials in response to current injection. Signals were filtered at 1 kHz and sampled at 10 kHz. Data were collected and analyzed using pCLAMP 10 software (Molecular Devices, CA).

### Treatments

A known amount of cocaine hydrochloride was dissolved in phosphate-buffered saline (PBS) as 1 M stock just prior to the assays. In order to simulate in vivo pharmacological concentrations of cocaine, ranging from 1 nM to 6.25 μM, several working stocks (0.04, 0.2, 0.4, 1, 2, 4, 8, 20, 40, 200, and 400 μM) were prepared in PBS; similarly, for higher cocaine concentrations (2, 3, and 4 mM final), working stocks of 80, 120, and160 mM were prepared in PBS. From each working stock, 5 μl cocaine was added per well to achieve desired concentrations as well as to prevent pH alterations in culture medium^37^. Final volume in each well was 200 μl. While the selection of lower concentrations was based on the reports of nano to lower micro molar cocaine in CNS cells of addicts^13^, the higher concentrations were based on several in vitro studies^14–16^. Cells with medium alone or equal volume of vehicle (PBS) in medium served as controls, while medium devoid of cells was taken as a blank. Based on our previous studies in C6 astroglia-like cells^6^, cocaine treatments in the present study were also carried out for 1 h for comparison purposes. Incidentally, cocaine effect in addicts wears off within this period for typical amounts and routes of intake^33^. In a subset of experiments, the cells in 96-well plates were pre-treated with 1 µM YM155, an inhibitor of *survivin* gene, for 30 min, followed by cocaine (2–4 mM) co-treatment for 1 h and evaluated for cell viability, while in other studies, the cells were pretreated with 5 μM PIK-75, an inhibitor of *Nrf-2*^24^, for 30 min prior to cocaine co-treatment (2–4 mM) for 1 h and evaluated for cell viability and GSH levels.

### Viability and vacuolation

Cell viability was assessed by crystal violet dye-uptake as described previously^54,55^. The extent of vacuolation in cells was quantified in glutaraldehyde fixed cells by neutral red dye uptake as described earlier^56^. The dye was extracted with 70% ethanol and 0.37% hydrochloric acid, and the absorbance at 540 nm was taken in a plate reader. Crystal violet stained cells were used for photomicrograph of vacuoles using an inverted phase contrast IX-70 Olympus microscope with a ×40 objective.

### Video microscopy

For live videography, one of the ocular lenses of the inverted phase contrast IX-70 Olympus microscope was replaced with a standard ocular video-camera system. The outlet of the video connector was attached to a computer for image visualization on the monitor using the Electronic Ocular -R-AMCap software program, supplied by Zhejiang JinCheng Scientific & Technology Co., Ltd, (HangZhou, China). Video documentation was taken at a speed 14 frames per second.

### Cell membrane integrity assay

Cell membrane integrity was determined by measuring the release of cytoplasmic LDH with CytoTox 96 non-radioactive assay kit (Promega, Madison, WI) as per the instructions provided by the manufacturer. In brief, the cells in 96-well microtiter plates were treated with various concentrations of cocaine (2, 3, and 4 mM) for 1 h. Then 50 μl of test medium was transferred into a new 96-well plate, mixed with equal volume of substrate mix from the kit, and incubated for 30 min at 37 °C. Absorbance was taken in a plate reader at 490 nm.

### Measurement of intracellular ROS

Cells in 96-well plates were treated with cocaine at 2, 3, and 4 mM for 1 h, followed by staining with a cell permeable dye H_2_DCFDA (10 μM final) for 30 min^6^. After gentle washing and air drying of the cells, PBS (100 μl/well) was added. The plates were read with the excitation filter set at 485 nm and the emission filter at 530 nm in an automatic reader (BioTek™ Synergy HTX multimode micro plate reader, BioTek Instruments, Winooski, VT).

### Lipid peroxidation assay

Cells were seeded at a starting density of 0.3 × 10^6^ cells per well in six-well plates. Lipid peroxidation was measured as described earlier^57^. In brief, after cocaine treatment at 2, 3, and 4 mM for 1 h, the cells were harvested and centrifuged at 13,000 rpm on a table top micro centrifuge for 6 min. All cell pellets were sonicated in PBS on ice for 3 s and transferred into glass tubes. Then the lysates were mixed with 30% trichloroacetic acid, 0.37% thiobarbituric acid (TBA). The mixture was boiled for 10 min, cooled to room temperature (RT), transferred into new falcon tubes and centrifuged at 3038 × *g* for 10 min. The clear supernatant was transferred into 96-well plate and absorbance at 535 nm was measured in a micro plate reader. Clear medium without cells was used as a blank.

### General mitochondrial metabolic activity and membrane potential

Cells (2 × 10^4^) were treated with various concentrations of cocaine (2, 3, and 4 mM) for 1 h in 96-well microtiter plates. Then the plates were centrifuged at 304 × *g* for 4 min, and the cocaine containing medium was discarded carefully. After adding fresh medium to cells (200 μl) immediately, 10 μl of MTS (3-(4,5-dimethylthiazol-2-yl)-5(3-carboxymethonyphenol)-2-(4-sulfophenyl)-2H-tetrazolium, Promega) was added per well as reported earlier^58^ and incubated for 30 min at 37 °C. Absorbance was taken in a micro plate reader at 490 nm. Membrane potential was assayed as per the earlier procedure^46^. At the end of 1 h treatment with cocaine, the monolayer cells were overlaid with 100 μl of 0.25% aqueous glutaraldehyde for fixation, containing Rh 123 to yield a final concentration of 2.6 μM for 30 min at RT. The supernatant was discarded, and the plates were washed with water and air dried in the hood. Finally, 100 μl of 0.1% Triton×100 in PBS was added per well and incubated at 37 °C for 1 h. The plates were read with the excitation filter set at 485 nm and the emission filter at 538 nm on a BioTek™ Synergy™ HTX multi-mode micro plate reader.

### Lactate detection

Studies conducted on cells grown in medium containing 10% FBS gave high background values due to serum interaction with some of the kit components (Trinity Biotech, Jamestown, NY). So, in our subsequent studies, prior to the treatments, 10% FBS containing medium was replaced with reduced serum (0.1% FBS) in 96-well microtiter plates (2 × 10^4^ cells per well). The kit reagent was dissolved in chromogenic solution that consisted of 5.3 mM vanillic acid, 2.9 mM 4-amino antipyrine, and about 4 units of horseradish peroxidase. At the end of 1 h cocaine treatment, the kit reagent was added directly to the wells (20 μl per 200 μl) and the plates were kept in the incubator at 37 °C for color development (5–10 min). Absorbance from the untreated cells was taken as a control, while the medium devoid of cells was taken as a blank. Absorbance was measured at 490 nm in a micro plate reader.

### NMR spectroscopy

Lactate release from the cells was detected by proton (^1^H^+^) NMR spectroscopy. Since the amount of serum does not interfere in this method, we used 10% FBS in medium in 96-well microtiter plates (2 × 10^4^ cells/200 μl per well). At the end of 1 h cocaine treatment, medium (0.15 ml per well) from all replicates (*n* *=* 12) of each treatment was pooled (1.8 ml) in the labeled tubes. Medium from the untreated cells was used as a control. Because the treated samples contained 2–4 mM cocaine, we added cocaine (4 mM final) to the media blank. NMR analyses were carried out on Bruker Avance 800 ($\nu _0$[1 H] = 800.23 MHz) that is equipped with a TCI 800S6 H-C/N-D-05 Z cryoprobe (the maximum *z*-field gradient strength is 48 G/cm). For obtaining one-dimensional (1D) NMR spectrum, 16 transients were acquired and co-added with 3 s of acquisition delay. Data points (32,000) were employed for obtaining a spectral width of 7500 Hz. The dominant water peak positioned at 4.75 ppm in the spectrum was eliminated by using the WATERGATE W5 pulse sequence^20^. A bandwidth of 3000 Hz along each side of the water peak was given as a spectral window for observing solute peaks by employing a delay time of 333.3 μs for the WATERGATE pulse trains. An external standard, 3.3 mM aqueous DMSO solution, was prepared for the quantification of the lactate in the sample solution.

### Measurement of H_2_S in cell culture medium

Cells were seeded at a starting density of 2 × 10^4^ per well in 96-well plates. Prior to cocaine treatment, 1.64% aqueous zinc acetate^59,60^ (final: 0.041%) was added to cells to convert volatile H_2_S gas into zinc sulfide during cocaine treatment. After 1 h of treatment with 2, 3, and 4 mM cocaine, 17.453 mM N,N-dimethyl-*p*-phenylenediamine sulfate in 7.2 M HCl (final: 2.55 mM) and 26.18 mM FeCl_3_ in 1.2 M HCl (final: 3.83 mM) were added to the wells and vortexed gently. Bubbles in medium were removed by adding 5 μl of cold ethanol to all wells just before reading the plates at 670 nm in a micro plate reader.

### ATP bioluminescence assay

Cells in 96-well microtiter plates were treated with various concentrations of cocaine (2, 3 and 4 mM) for 1 h. Total ATP was measured using Bioluminescent ATP Somatic cell assay kit (FLASC, Sigma–Aldrich) as per the kit instructions supplied by the manufacturer. In brief, at the end of treatment, 75 μl of Somatic Cell ATP releasing solution was added per well to lyse the cells. Then 50 μl lysate was transferred into new white, clear bottom 96-well plates, which already contained 50 μl of ATP assay mix enzyme under reduced light. Luminescence was measured immediately on a BioTek™ Synergy™ HTX multi-mode micro plate reader.

### Isolation of RNA and cDNA synthesis

The cells were seeded at a density of 2 × 10^6^ in 100 mm diameter culture dishes. At the time of treatment, the cells reached around 65–70% confluence. After 1 h treatment with 2–4 mM cocaine, the cells were harvested by scrapping and centrifuged at 1125 × *g* for 5 min. Total RNA was isolated as per the protocol supplied by the manufacturer (Qiagen Sciences, German town, MD) using RNeasy mini spin columns. DNase I (Qiagen Sciences) treatment was performed on the column. Total RNA from the column was eluted with 20 μl of RNase free water. For the sake of quantification, the RNA on ice was diluted in RNase free water at 1:10 ratio. The quantity of total RNA was measured by the Nanodrop ND-1000 spectrophotometer (NanoDrop Technologies, Wilmington, DE). The RNA showed a ratio of >1.95 at 260/280 nm, and was subsequently used for cDNA synthesis. One microgram of total RNA was used to produce cDNA using an iScript cDNA synthesis Kit (Bio-Rad, Hercules, CA) according to the manufacturer’s instructions.

### Relative expression by quantitative RT-PCR

Gene expression analysis of *Nrf-2*, *survivin* (Birc5), and *GAPDH* by qPCR was performed in an iCycler thermal cycler with MyiQ detection system (Bio-Rad) using iQ SYBR Green supermix. *Nrf-2*, *survivin*, and *GAPDH* products were synthesized by separate PCR reactions, carried out in 20 μl final volume with 2 μl of cDNA sample and 500 nM of specific primers. Cycling conditions were as follows: 95 °C for 10 min, followed by 40 cycles of 95 °C for 15 s, 55 °C for 30 s and 72 °C for 30 s, while melt curve analysis following PCR was performed at 55–95 °C. The relative quantification of gene expressions was performed according to 2^−△△CT^ method^61^ with *GAPDH* as the endogenous control. The primer sequences used for analyses are shown in Table 1.

**Table 1:** Primer sequences

Gene name	Accession/nucleotide position	Primers	Fragment length
*Nrf-2*	NCBI:NM 010902.4 (Nucleotides: 1468–1488)	5′-CACAGTGCTCCTATGCGTGAA-3′ 5′-TTTGTGAATGGGGCTTTTTGA-3′	92 bp
*Survivin* (Birc5)	NCBI:NM 009689.2 (Nucleotides: 492–512)	5′-GACTGCAAAGACTACCCGTCA-3′ 5′-GATGTGGCATGTCACTCAGG-3′	94 bp
*GAPDH*	NCBI:NM 001289726.1 (Nucleotides: 832–853)	5′-TGGAGAAACCTGCCAAGTATGA-3′ 5′-TGGTCCTCAGTGTAGCCCAAG-3′	94 bp

### Total GSH assay

After treating with different concentrations of cocaine (2–4 mM) in complete medium for 1 h in 96-well microtiter plates, the cells were fixed with 0.25% glutaraldehyde for 30 min, followed by gentle washing three times, and air dried. Total cellular GSH was assayed as described earlier^62^. The absorbance was measured at 412 nm in a micro plate reader.

### Preparation of whole-cell lysates

For enzyme assays, cells were seeded at a density of 2 × 10^6^ in 100 mm diameter culture dishes. After 1 h treatment with 2–4 mM cocaine, the cells were harvested using cell scrappers. After centrifugation at 1620 × *g* for 5 min, the cell pellets were stored at −70 °C until further use. On the day of assays, the pellets were re-suspended in 0.5 ml PBS and sonicated twice on ice for 15 s. The contents were transferred to eppendorf tubes and microcentrifuged at 5000 rpm for 5 min. The supernatants were transferred to new tubes and used for enzyme assays. Protein concentration in each lysate was determined using BCA protein assay kit (Pierce, Rockford, IL) as per manufacturer’s instructions taking bovine serum albumin as a standard reference.

### Catalase assay

It was assayed as per the earlier method^63^. The reaction mixture (450 μl) in a quartz cuvette contained 50 μl of cell lysate (60 μg), 250 μl of 50 mM phosphate buffer (pH 7.0). The reaction was started by the addition of 150 μl of 30 mM H_2_O_2_ (10 mM final). The decrease in absorbance at 240 nm was monitored for 2 min in a Genesys 10 S UV-Vis spectrophotometer (Thermo Scientific, Vernon Hills, IL). The enzyme activity was calculated using the extinction coefficient of 0.00706 per mmol per mm and the unit of enzyme activity was expressed as mmol H_2_O_2_ decomposed per minute per mg protein. Then the enzyme activity of treated samples was compared with the control (100%).

### GPx assay

It was assayed as per the published procedure^64^. The reaction mixture (500 μl) contained 1 mM GSH, 0.15 mM NADPH, 0.12 unit glutathione reductase (GR), 0.1 mM sodium azide in 0.1 M potassium phosphate (pH 7.0 with 1 mM EDTA. Sodium azide was added to the reaction mixture to inhibit endogenous catalase activity. The reaction mixture was incubated with 60 μg of sample for 10 min without NADPH, and the reaction was started by the addition of NADPH and H_2_O_2_ at a final concentration of 150 μM. The rate of NADPH consumption was monitored at 340 nm for 3 min. One unit of GPx activity was defined as the amount of enzyme required to consume 1 μmol of NADPH/min in the coupled assay and activity was expressed per mg of protein. Then the enzyme activity of treated samples was compared with the control (100%).

### Statistical analysis

The experimental results (*n* = number of wells pooled from at least two different experiments) were presented as the mean ± SEM. All bar graphs were plotted using a GraphPad Prism Software, version 3.00 (San Diego, CA). The data were analyzed for significance by one-way ANOVA and then compared using Dunnett’s or Bonferroni’s multiple comparison tests. The test values of *P* *<* 0.05 and *P* < 0.01 were considered significant and highly significant, respectively.

## Electronic supplementary material

Figure S3

Figure S4

Supplementary Text

Supplementary material

Neuronal-like morphology of N2a cells

Electrophysiological properties of N2a cells

C6 cells

N2a cells
