# Supplementary figures and images for: Cellular and molecular responses to acute cocaine treatment in neuronal-like N2a cells: potential mechanism for its resistance in cell death
Source: Cell Death Discov. 2018 Jul 17;4:76. doi: 10.1038/s41420-018-0078-x (PMC6133924; doi:10.1038/s41420-018-0078-x)

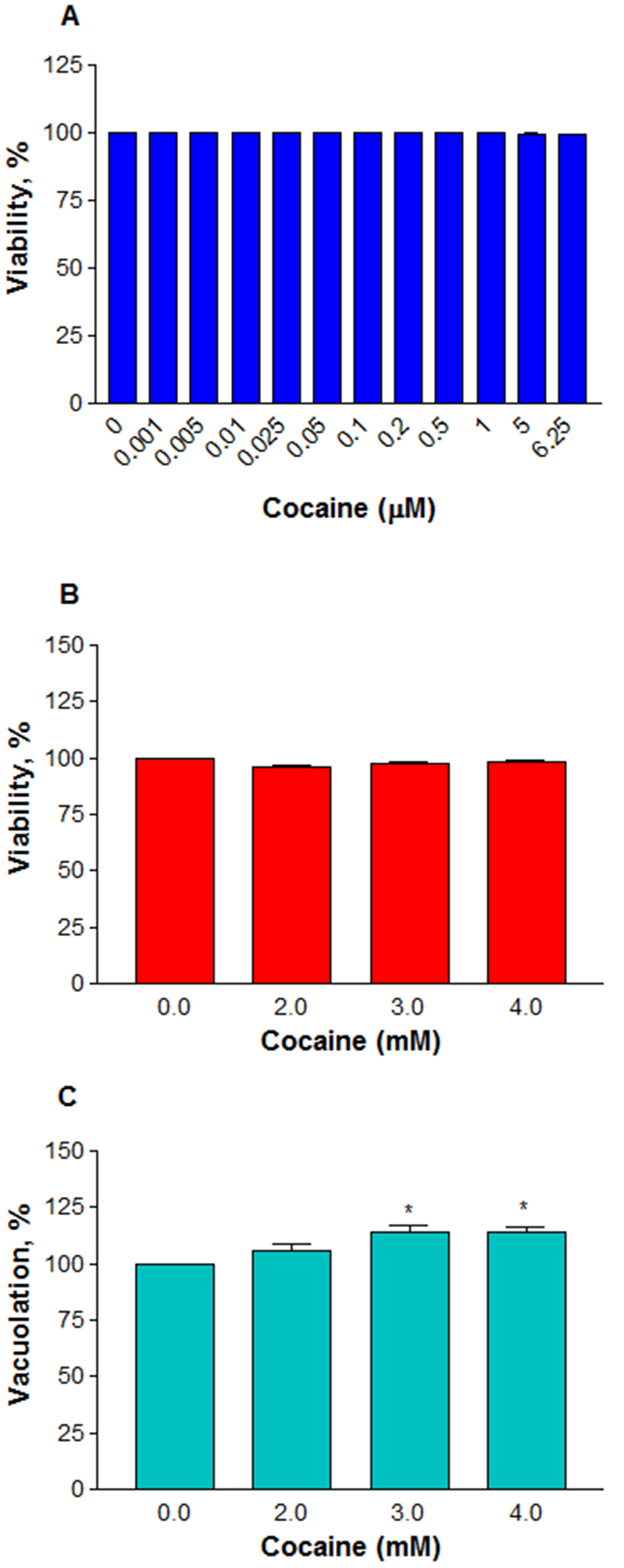

Supplement: Supplementary file 1 — Figure S3 [file 41420_2018_78_MOESM1_ESM.tif]

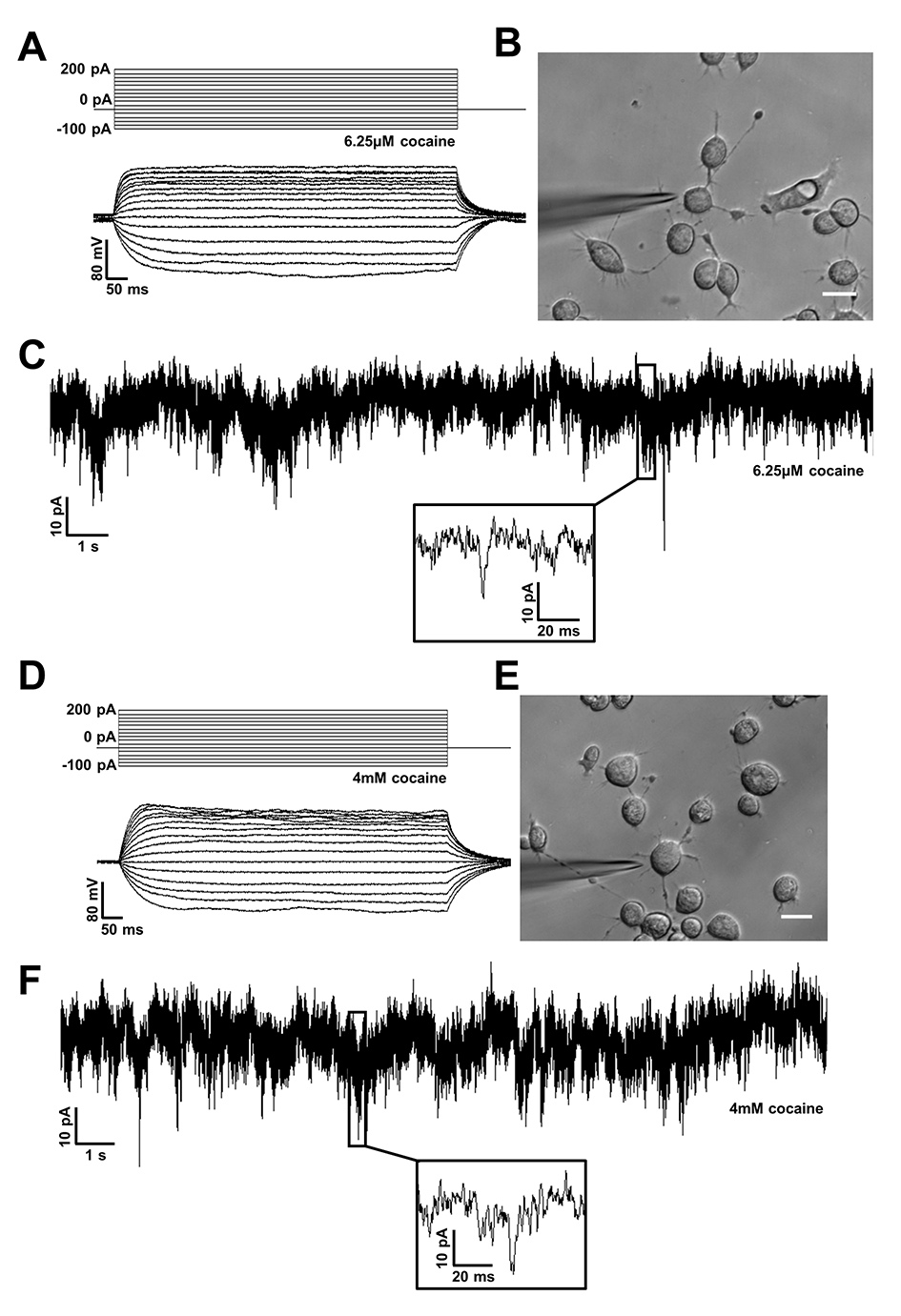

Supplement: Supplementary file 2 — Figure S4 [file 41420_2018_78_MOESM2_ESM.tif]

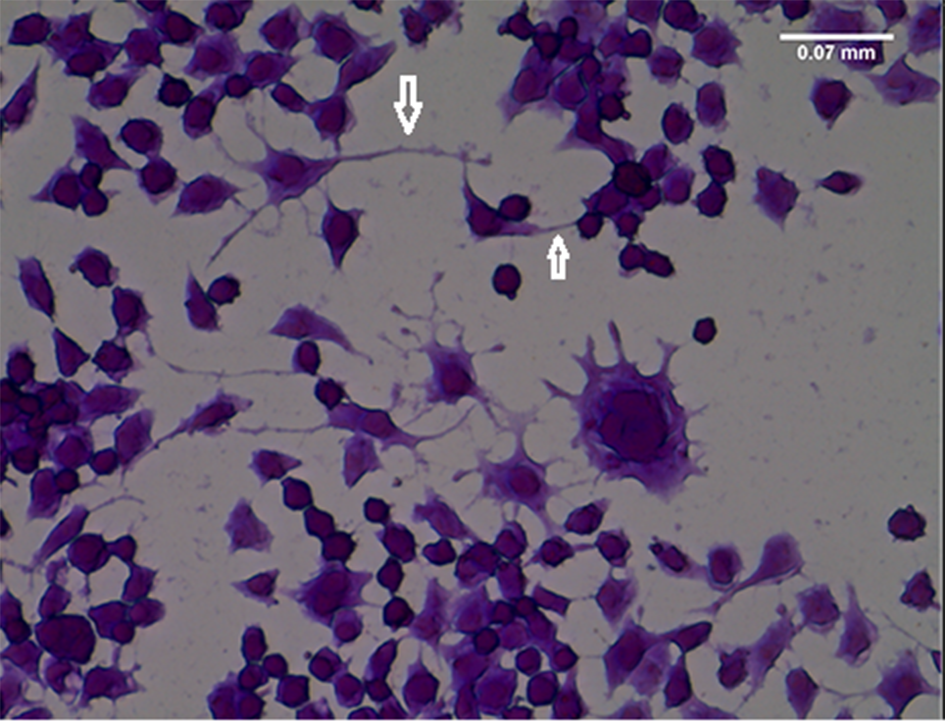

Supplement: Supplementary file 5 — Neuronal-like morphology of N2a cells [file 41420_2018_78_MOESM5_ESM.tif]

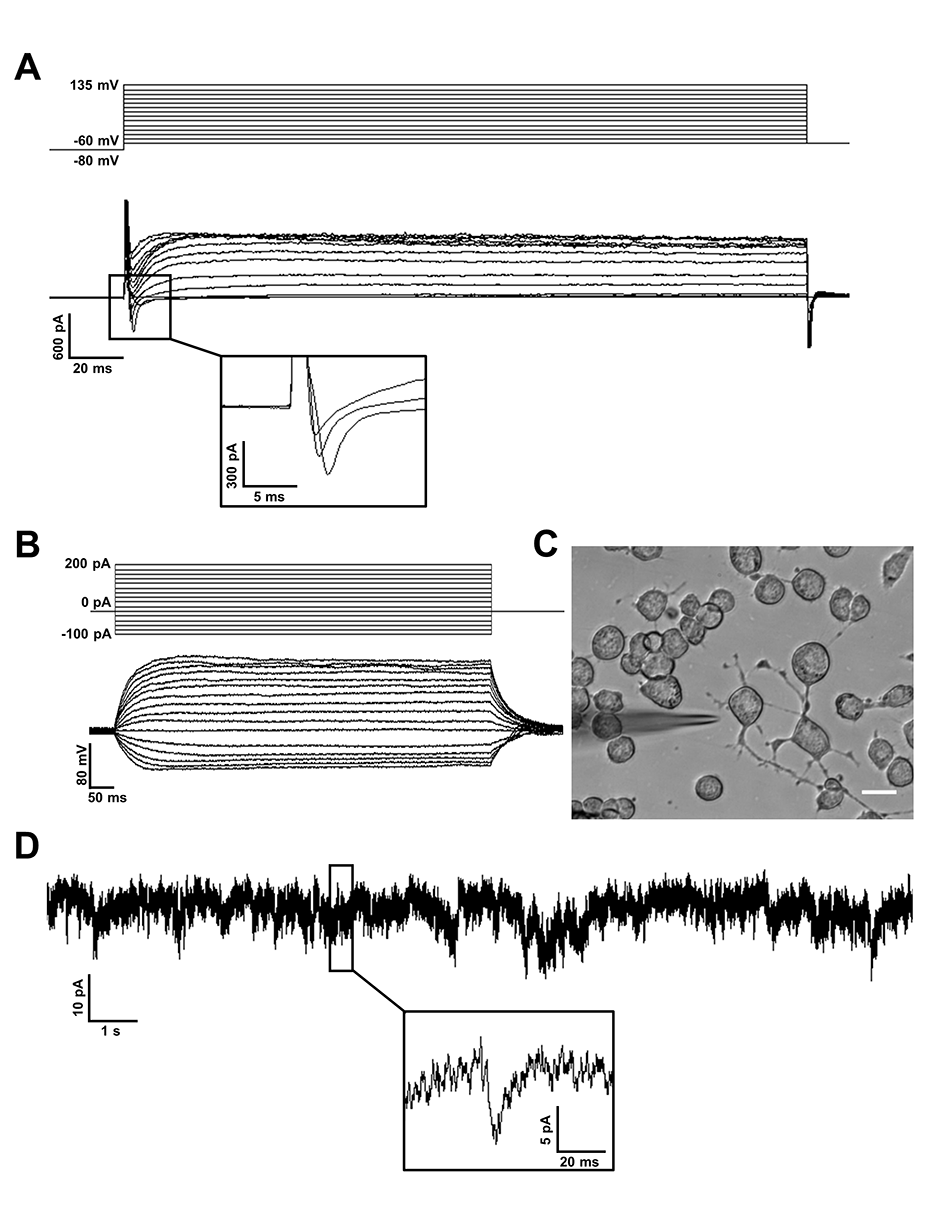

Supplement: Supplementary file 6 — Electrophysiological properties of N2a cells [file 41420_2018_78_MOESM6_ESM.tif]
