# Supplementary material for: Cellular and molecular responses to acute cocaine treatment in neuronal-like N2a cells: potential mechanism for its resistance in cell death
Source: Cell Death Discov. 2018 Jul 17;4:76. doi: 10.1038/s41420-018-0078-x (PMC6133924; doi:10.1038/s41420-018-0078-x)
Supplement: Supplementary file 3 — Supplementary Text [file 41420_2018_78_MOESM3_ESM.doc]

**Supplementary data**

**Methods**

**Morphology and electrophysiology**

For gross cell morphological evaluations, crystal violet stained cells (Badisa et al., 2010) were photomicrographed using EVOS Cell Imaging Systems with 40x objective. For electrophysiology study, the same experimental procedure described in the methods section of the main text was followed.

**Supplementary results**

**Neuronal characteristics of N2a cells**

Under our experimental conditions, some population of N2a cells in the absence of external growth factors had undergone various degrees of spontaneous differentiation after 4-5 days of growth in the incubator and acquired neuronal morphology (Supplementary Figure S1). The neurites branched out from the cell bodies and connected to the neurites of the neighboring cells, forming intercellular junctions. The average neurite extension per cell was found to be 7.4 microns; the minimum was 34 microns while the maximum was 131 microns. To further assess neuronal characteristic of the cells, electrophysiological properties were measured by whole-cell patch clamp method. Differentiated cells (Supplementary Figure S2C) displayed fast inward currents and long-lasting outward currents in response to depolarizing voltage steps, indicative of functional voltage-gated Na^+^ and K^+^ channels, respectively (Supplementary Figure S2A). While current injection failed to elicit action potentials, spontaneous postsynaptic currents were observed in the absence of stimulation (Supplementary figures S2B and S2D). The presence of these sub-threshold level synaptic events demonstrated that N2a cells are electrically active in culture, despite their lack of spike activity. Taken together, these results suggest that N2a cells exhibited neuron‑like characteristics both morphologically and functionally. No differences were observed between untreated and cocaine treated cells in spike activity or spontaneous postsynaptic current activity (Supplementary Figure S3).

**Justification of high cocaine concentrations**

The use of higher cocaine doses in our study became imperative owing to lack of effect on morphology (Fig. 1A) or viability (Fig. 1B) at lower concentrations. This necessitated increasing of cocaine concentrations several fold. Under *in vitro* studies, cocaine concentrations have extended into milli molar range^1-4^. Thus testing 8.8 mM^5^ or 10 mM^1,4,6^ or 13 mM^7^ cocaine at even longer incubation intervals despite cocaine's relatively short half-life of ~1 hour^8-10^ are not atypical. The highest concentration (4 mM) in our study was 2.2 to 3.25 times lower compared to 8.8 mM^5^ and 13 mM^7^ cocaine, respectively. Therefore, cocaine concentrations tested in our study were in the acceptable range for *in vitro* research. On the other hand, nano to micro molar^11^ cocaine to elicit pharmacological response in human addicts do not necessarily reflect the actual amount of cocaine intake by them, because the assessment to determine these levels does not take into consideration of drug tolerance or its frequent use by addicts^12^ or cocaine hydrolysis by blood esterases^13,14^. A well-adapted abuser can take in as much as 5 g of cocaine per day^15^; this roughly translates to 3 mM (MW: 339.8) assuming a total blood volume of 5 liters in an average human being; or 7.7 mg of cocaine by a juvenile-adult rat (120 g weight and 64 ml blood/Kg body weight) or 64 mg/Kg. Since cocaine in animal model could be administered as much as 100 mg/Kg or more per day^16^, the concentrations in our study were within the realm of *in vivo* experiments. The clinical use of cocaine as a local anesthesia by medical practitioners is typically at 10%, which is equal to 294.2 mM, while in our case, the concentrations of 2-4 mM represent 0.068 to 0.1361% only, a 7 to 14 fold less compared to anesthesia dose. Thus considering the *in vitro* or *in vivo* or clinical doses of cocaine, our concentrations fall within the acceptable range of research.

**Supplementary references**

1. Repetto G, del Peso A, Garfia A, et al. Morphological, biochemical and molecular

effects of cocaine on mouse neuroblastoma cells culture in vitro. *Toxicol In Vitro*

1997;11:519-525.

2. Oliveira, M. T., Rego, A. C., Morgadinho, M. T., Macedo, T. R., and Oliveira, C. R. (2002). Toxic effects of opioid and stimulant drugs on undifferentiated PC12 cells. *Ann. N. Y. Acad. Sci*. 965, 487-496.

3. Cunha-Oliveira, T., Rego, A. C., Cardoso, S. M., Borges, F., Swerdlow, R. H., Macedo, T., et al. (2006a). Mitochondrial dysfunction and caspase activation in rat cortical neurons treated with cocaine or amphetamine. *Brain Res*. 1089, 44-54.

4. Cunha-Oliveira, T., Rego, A. C., Morgadinho, M. T., Macedo, T., and Catarina Resende- Oliveira, C. T. (2006b). Differential cytotoxic responses of PC12 cells chronically exposed to psychostimulants or to hydrogen peroxide. *Toxicology* 217, 54-62.

5. Yu RCT, Lee TC, Wang TC, et al. Genetic toxicity of cocaine. *Carcinogenesis*

1999;20(7):1193-1199.

6. Lattanzio F.A, Tiangco D, Osgood C, Beebe S, Kerry J, and Hargrave B.Y (2005) Cocaine increases intracellular calcium and reactive oxygen species, depolarizes mitochondria, and activates genes associated with heart failure and remodeling. Cardiovasc Toxicol 5:377–389.

7. Kugelmass AD, Oda A, Monahan K, et al. Activation of human platelets by cocaine.

*Circulation* 1993;88:876-883.

# 8. [**Wilkinson, P**](https://www.ncbi.nlm.nih.gov/pubmed/?term=Wilkinson P%5BAuthor%5D&cauthor=true&cauthor_uid=7357795)., [**Van Dyke, C**](https://www.ncbi.nlm.nih.gov/pubmed/?term=Van Dyke C%5BAuthor%5D&cauthor=true&cauthor_uid=7357795)., [**Jatlow, P**](https://www.ncbi.nlm.nih.gov/pubmed/?term=Jatlow P%5BAuthor%5D&cauthor=true&cauthor_uid=7357795)., [**Barash, P**](https://www.ncbi.nlm.nih.gov/pubmed/?term=Barash P%5BAuthor%5D&cauthor=true&cauthor_uid=7357795)., and [**Byck, R**](https://www.ncbi.nlm.nih.gov/pubmed/?term=Byck R%5BAuthor%5D&cauthor=true&cauthor_uid=7357795). (1980). Intranasal and oral cocaine kinetics. [***Clin. Pharmacol. Ther*.**](https://www.ncbi.nlm.nih.gov/pubmed/7357795) 27(3), 386-94.

9. Barnett, G., Hawks, R., and Resnick, R. (1981). Cocaine pharmacokinetics in humans. *J. Ethnopharmacol*. 3, 353–366.

10. Chou MJ, Ambre JJ, Ruo TI: Kinetics of cocaine distribution, elimination, and chronotropic effects. Clin Pharmacol Ther 38:318-324, 1986

11. Zheng, F., and Zhan, C. G. (2012). Modeling of pharmacokinetics of cocaine in human reveals the feasibility for development of enzyme therapies for drugs of abuse. *PloS Comput. Biol*. 8, e1002610. doi: 10.1371/journal.pcbi.1002610 PMID: 22844238

12. Stichenwirth M, Stelwag-Carion C, Klupp N, et al. Suicide of a body packer.

*Forensic Sci Int* 2000;108:61-66.

# 13. Spiehler V.R, Reed D (1985) Brain concentrations of cocaine and benzoylecgonine in fatal cases. J Forensic Sci 30(4):1003-1011.

14. Fraker TD Jr, Temesy-Armos PN, Brewster PS, et al. Mechanism of cocaine-induced

myocardial depression in dogs. *Circulation* 1990;81:1012–1016.

15. Stewart A, Heaton ND, Hogbin B. Body packing--a case report and review of the

literature. *Postgrad Med J* 1990;66:659-661.

16. Ryan, L. J., Martone, M., Linder, J., and Groves, P. M. (1988). Cocaine, in contrast to d-amphetamine, does not cause axonal terminal degeneration in neostriatum an agranular frontal cortex of Long–Evans rats. *Life Sci*. 43, 1403-1409.

**Supplementary figure legends**

**Supplementary Fig. 1. Neuronal-like morphology of N2a cells.** Cells were stained with crystal violet and optical images were taken using EVOS Cell Imaging Systems with 40x objective. Arrows indicate spontaneously differentiated neurites. Scale bar: 0.07 mm.

**Supplementary Fig. 2. Electrophysiological properties of N2a cells.** (**A**) Representative voltage clamp trace showing fast inward currents and long-lasting outward currents evoked by depolarizing voltage steps. Step size = 15 mV. The inset shows a high magnification view of the inward currents. (**B**) Representative current clamp trace showing that N2a cells lack spiking activity in response to current injections. Step size = 20 pA. (**C**) Representative phase-contrast images of a recorded cell. Scale bar = 20 μm. (**D**) Representative trace of continuous voltage clamp recording showing spontaneous postsynaptic currents. The inset shows a high magnification view of one spontaneous postsynaptic current.

**Supplementary Fig. 3. Effect of cocaine on viability and vacuolation**. The cells were treated with equal volume of vehicle (PBS control) or cocaine (0.001 to 6.25 μM or 2-4 mM) for 1 h. Cell viability (**A**, *n* **=** 8; **B,** *n* **=** 12) or vacuolation (**C**, *n* = 12) was measured in a micro plate reader. Data were represented as mean ± SEM, *P* > 0.05, insignificant or **P* < 0.05, significant compared to control, one-way ANOVA, Dunnett’s multiple comparison test.

**Supplementary Fig. 3. Electrophysiological properties of cocaine-treated N2a cells**. Spiking activity, cellular morphology, and spontaneous postsynaptic currents are unaffected by low dose (**A-C**) and high dose (**D-F**) cocaine treatment. (**A and D**) Representative current clamp traces showing lack of spiking activity in response to current injections. Step size = 20 pA. (**B and E**) Representative phase-contrast images of a recorded cell. Scale bar = 20 μm. (**C and F**) Representative traces of continuous voltage clamp recording showing spontaneous postsynaptic currents. The inset shows a high magnification view of one spontaneous postsynaptic current.
